# Prevalence of antibiotic-resistant bacteria amongst dogs in Africa: A meta-analysis review

**DOI:** 10.4102/ojvr.v89i1.1970

**Published:** 2022-10-10

**Authors:** Ayaovi B. Yaovi, Philippe Sessou, Aretas B.N. Tonouhewa, Gildas Y.M. Hounmanou, Deborah Thomson, Roger Pelle, Souaïbou Farougou, Arindam Mitra

**Affiliations:** 1 Research Unit on Communicable Diseases, Polytechnic School of Abomey-Calavi, University of Abomey-Calavi, Cotonou, Benin; 2 Department of Veterinary and Animal Sciences, University of Copenhagen, Copenhagen, Denmark; 3 One Health Lessons, Arlington, Virginia, United States of America; 4 International Livestock Research Institute, Nairobi, Kenya; 5 Department of Microbiology, Adamas University, Kolkata, West Bengal, India

**Keywords:** prevalence, antibiotic resistance, dogs, Africa, meta-analysis, antimicrobial resistance

## Abstract

Antimicrobial resistance (AMR) is a global public health threat for both human and veterinary medicine. Increasing evidence suggests that animals are important sources of AMR to humans; however, most of these studies focus on production animals. In order to determine the pattern of AMR in pets, mainly in dogs in Africa, a meta-analysis was performed with AMR studies conducted in African countries and published between January 2000 and January 2021 in four databases: Medline (PubMed), Scopus, Cab abstract and Google Scholar. Seven bacterial strains, namely *Staphylococcus aureus, Escherichia coli, Salmonella* spp., *Pseudomonas aeruginosa, Streptococcus pyogenes,* coagulase-negative *Staphylococcus* (SNC) and *Staphylococcus pseudintermedius* were included in this study. A total of 18 out of 234 indexed articles met the study criteria. The results revealed that multiple bacteria were resistant to various commonly used antibiotics including enrofloxacin, ciprofloxacin, gentamicin, amoxicillin, clavulanic acid, cotrimoxazole, streptomycin, tetracycline and chloramphenicol. Concerning multidrug resistance, *E. coli* strains came first with the highest prevalence of 98%, followed by *P. aeroginosa* (92%) and *Salmonella* spp. (53%). In contrast, the overall prevalence of multidrug resistance was low for *S. aureus* (18%) and *S. pseudintermedius* (25%). It is therefore urgent to find, as soon as possible, alternatives to replace these antibiotics, which have become ineffective in controlling these bacteria in dogs in Africa. Moreover, further metagenomic studies are needed to describe the full resistome and mobilome in dogs regardless of the bacteria.

## Introduction

The importance of the bond between people and their pets is increasingly recognised and many owners consider their pets to be a member of their family (WSAVA 2020). This is particularly the case with dogs that are highly valued amongst all other pets because of their many benefits to humans such as sheep guarding, detection of drugs and explosives, hunting and safety, breeding and companionship (Daodu et al. 2017). For years, the dog was the most widely employed scent-detector tool for civilian and military purposes. Recently, many studies highlighted the role of canine olfactory ability in the medical field, specifically in detecting different infectious, metabolic and neoplastic conditions including the coronavirus disease 2019 (COVID-19) (Sakr et al. 2021). Trained biodetection dogs are already being used for detecting illicit substances and for forensic purposes. They have also been used for helping to detect cancer (Sharun et al. 2021). A study conducted by Oliva and Johnston (2020) found a buffering effect of dog ownership against loneliness. Despite the benefits of dog ownership, dogs are susceptible to many infectious agents responsible for viral diseases such as rabies, parvovirosis, canine distemper and bacterial diseases, namely leptospirosis, pasteurellosis, skin diseases and parasitic illness such as piroplasmosis, and so on (Ghasemzadeh & Namazi 2015). As a result of their importance, most owners try to take care of their animals to the best of their ability and strive to keep their animals healthy and ‘happy’ (WSAVA 2020). This has led to a high use of antimicrobials such as antibiotics to treat diseases and injuries in their animals and the use of vaccines for the prevention of certain diseases (Gwenzi et al. 2021). Antibiotic resistance is a global public health problem that could be responsible for more than 10 million deaths per year and thus become the leading cause of mortality by 2050, with an economic cost of $100 billion if left unchecked (O’Neill 2016). The antibiotic agents used are often more closely related to those used in human medicine and antibiotic resistance risks should not be ignored as the close relationship between dog and human subjects presents an opportunity for the two-way transfer of bacterial (commensal and pathogen) or genetic determinants of resistance with associated potential for morbidity and mortality on both sides (Argudín et al. 2017; Rendle & Page 2018). The misuse of antibiotics has been reported in several regions of the world and is at the origin of the antibiotic resistance phenomenon (Kavanagh, Mitra & Basu 2021; Le Huy et al. 2020). In Africa, numerous recent studies in different countries have revealed high prevalence of resistant bacterial strains in dogs (Qekwana et al. 2020; Zewdu et al. 2019). A survey conducted in Nigeria revealed that 82% of dog owners had, at different times, body-to-body contact of less than 50 cm between their face and the dog’s body (Daodu et al. 2017). This close contact can promote the exchange of resistant pathogens via saliva, urine, faeces, aerosols, skin and thus amplify the phenomenon of antibiotic resistance in humans and exchange of antibiotic resistance between humans and dogs. Given the increase prevalence of antibiotic-resistant bacteria and the risk of bacterial transmission between dogs and their owners, it is important to understand the overall level of antibiotic resistance in the dog population. With this knowledge, veterinarians can then make recommendations to protect the health of both their patients and clients. This meta-analysis aims to assess the overall prevalence of antibiotic-resistant bacterial strains in dogs in Africa to develop and implement local and continental control programmes for drug-resistant bacterial strains and their spread.

## Methodology

### Literature search

A systematic search was conducted according to the Preferred Reporting Items for Systematic Reviews and Meta-Analysis (PRISMA) protocol items on the phenomenon of antibiotic resistance in companion animals in Africa between January 2021 and March 2021. Searches were conducted in four databases, Medline (PubMed), Scopus, Cab abstract and Google Scholar by indexing several keywords in the title and abstract of the articles stored in these different databases. The search terms included ‘Companion animals’ and ‘Antimicrobial resistance’ and ‘Africa’ or ‘dog’ and ‘Antimicrobial resistance’ and ‘Africa’ or ‘dog’ and ‘Antibacterial resistance’ and ‘Africa’ or ‘dog’ and ‘Antibiotic resistance’ and ‘Africa’ or ‘Multidrug resistance’ and ‘Africa’ or ‘dog’ and these term with each of 54 African countries. All studies (in English or French) on bacterial antimicrobial resistance (AMR) in dogs published between January 2000 and January 2021 were included.

### Data extraction

After excluding irrelevant studies (non-African countries, antibiotic resistance in other animal species) and duplicates, the remaining studies were fully read in order to extract the data needed for this review: name of the first author, year of publication, country where the study was carried out, sources of samples, sample size, methods of analysis (disc diffusion method or molecular method), number of positive cases, number of bacterial strains tested and prevalence of antibiotic resistant strains. Figure 1 presents the flow chart of the process of identifying studies.

**FIGURE 1:**
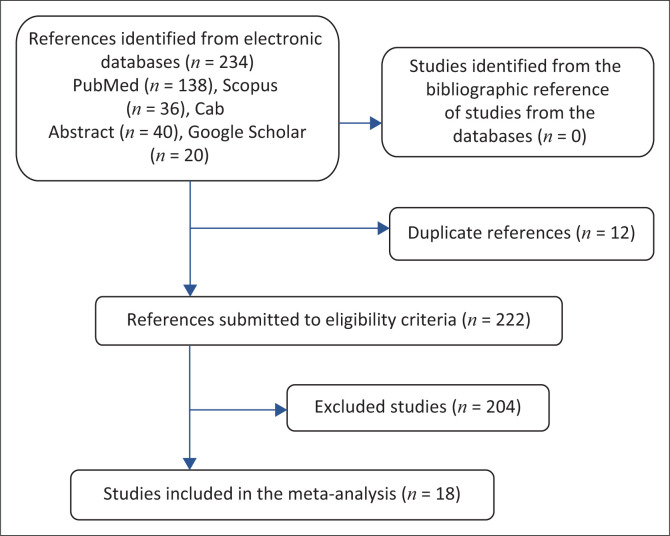
Workflow for selection of studies from various databases.

### Statistical analyses

Data extracted from all selected publications were directly inserted into the Excel spreadsheet and subjected to meta-analysis in R version 3.1 (R Core Team 2016) using the Meta and Metafor packages (Schwarzer 2007; Viechtbauer 2010). The Mantel-Haenszel fixed-effects model (FEM) and the Der Simonian and Laird random-effects model (REM) were used to generate an overall estimate of the prevalence of antibiotic resistance.

Heterogeneity between the different studies was assessed using the *I*^2^ statistic of Higgins et al. (2003). *I*^2^ values of 25%, 50% and 75% were considered to have a low, moderate and high degree of heterogeneity, respectively. When heterogeneity between studies is low, the fixed effect model estimates were considered, whilst the random effect model was used to generate the overall prevalence in case of high heterogeneity. Finally, to summarise the results of the meta-analysis, a forest plot showing the different prevalence estimates of multidrug resistance (MDR) bacteria across studies, a confidence interval and an overall prevalence was generated for each of the two models used.

### Ethical considerations

This article followed all ethical standards of research without direct contact with human or animal subjects.

## Results

### Characteristics of eligible studies

**FIGURE 2:**
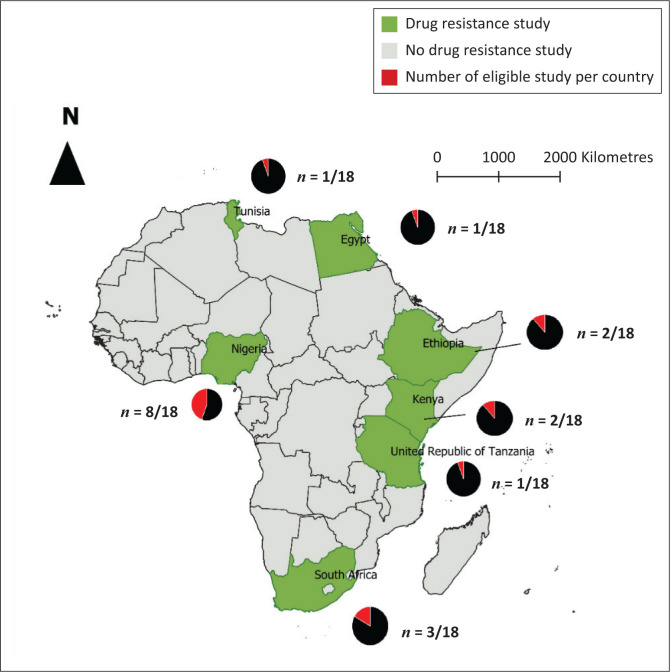
Map of countries where antibiotic resistance is studied amongst dogs.

The characteristics of the studies selected for the meta-analysis are presented in Table 1. A total of 18 eligible studies were selected for the meta-analysis out of 234 indexed articles. The selected studies took place in countries such as Nigeria (8 studies), South Africa (3), Kenya (2), Tanzania (1), Ethiopia (2), Egypt (1) and Tunisia (1) (Figure 2). The sources of contamination used in these studies are mainly, in order of importance, rectal, nasal, oral, skin, vaginal, ear canal and wound swabs. The methods used by these authors to study antibiotic resistance are disk diffusion and molecular methods.

**TABLE 1:** The outline of selected antimicrobial resistance studies in dogs.

Authors	Country	Year of publication	Methods used	Targeted species	Sources of bacteria	Number of dog samples	Number of samples collected
Mustapha et al. 2021	Nigeria	2021	Disc diffusion method	Dogs	Rectal swabbing	200	200
Eliasi et al. 2020	South Africa	2020	Disc diffusion method	Dogs	Skin, ear and urine swabbing	155	155
Zewdu et al. 2019	Ethiopia	2019	Disc diffusion method	Dogs	Rectal swabbing	438	438
Njoroge et al. 2018	Kenya	2018	Disc diffusion method	Dogs	Nasal, oral, perianal, wound and ear swabs	191	291
Qekwana et al. 2018	South Africa	2018	Disc diffusion method	Dogs	Urine	755	755
Anyanwu et al. 2017	Nigeria	2017	Disc diffusion method	Dogs	Rectal swabbing	100	100
Daodu et al. 2017	Nigeria	2017	Disc diffusion method	Dogs	Nasal swabbing	173	173
Kiflu et al. 2017	Ethiopia	2017	Disc diffusion method	Dogs	Rectal swabbing	360	360
Qekwana et al. 2017	South Africa	2017	Disc diffusion method	Dogs	Skin and ear canal swabbing	-	334
Bukar-kolo et al. 2016	Nigeria	2016	Disc diffusion method	Dogs, sheep, goats	Skin, vaginal and wound swabbing	15	-
Katakweba et al. 2016	Tanzania	2016	Disc diffusion method, PCR	Humans, pigs and dogs	Nasal swabbing	100	100
Mustapha et al. 2016	Nigeria	2016	Disc diffusion method, PCR	Dogs	Nasal and perineal swabbing	-	416
Awoyomi & Ojo 2014	Nigeria	2014	Disc diffusion method	Dogs	Buccal swabbing	62	62
Eze et al. 2014	Nigeria	2014	Disc diffusion method	Dogs	Vaginal swabbing	20	20
Ojo et al. 2014	Nigeria	2014	Disc diffusion method, PCR	Dogs	Rectal swabbing	94	94
Gharsa et al. 2013	Tunisia	2013	Disc diffusion method, PCR	Dogs	Nasal swabbing	100	100
Abdel-moein, El-Hariri & Samir 2012	Egypt	2012	Disc diffusion method, PCR	People, dogs and cats	Nasal, oral, wound and ear canal swabbing	70	70
Mande & Kitaa 2005	Kenya	2005	Disc diffusion method	Dogs	Swabbing of ear wounds and infections	78	78

### Prevalence of antibiotic-resistant bacterial strains isolated from dogs in Africa

Many bacterial strains isolated from dogs and identified either biochemically or genotypically have been tested for antibiotic efficacy in eligible studies, however only seven strains with major public health impacts are included in this meta-analysis. These are *Staphylococcus aureus, Escherichia coli, Salmonella* spp., *P.aeruginosa, Streptococcus pyogenes, coagulase-negative Staphylococcus* and *Staphylococcus pseudintermedius*.

### Prevalence of antibiotic-resistant *Staphylococcus aureus* strains

Table 2 shows the overall prevalence of antibiotic-resistant *S. aureus* isolates from dogs in Africa. Analysis of this table reveals a higher prevalence (> 52%) of *S. aureus* isolates resistant to the following antibiotics: nalidixic acid, streptomycin, methicillin, cotrimoxazole, ampicillin, amoxicillin, kanamycin, tetracycline, penicillin G and pefloxacin. These isolates showed a low prevalence of resistance to the other antibiotics tested such as oxacillin, gentamycin, enrofloxacin, norfloxacin, erythromycin and a higher sensitivity to ofloxacin and ciprofloxacin. The prevalence of multidrug resistant strains was 18%.

**TABLE 2:** The overall prevalence of antibiotic resistant *Staphylococcus aureus* strains isolated from dogs in Africa.

Families	Antibiotics	Overall prevalence† (%)	95% CI	*n*	*N*	Number of studies	*I* ^2^
Beta-lactams	Ampicillin	**73.40**	40.0–91.9	152	251	6	66
	Amoxicillin	**71.70**	64.5–78.0	122	170	2	62
	Amoxicillin-clavulanic acid	33.30	20.0–92.5	77	157	3	22
	Oxacillin	11.70	0.06–96.9	87	139	2	0
	Penicillin G	**56.23**	40.1–71.1	50	87	3	62
	Methicillin	**77.80**	42.10–94.4	7	9	2	0
Aminoglycoside	Streptomycin	**80.64**	19.9–98.6	23	50	4	54
	Gentamicin	15.41	33.3–49.1	123	360	9	91
	Kanamycin	**66.70**	33.3–88.9	6	9	1	-
Quinolones	Ciprofloxacin	1.84	1.0–25.1	6	183	6	61
	Enrofloxacin	42.90	14.37–77.02	3	7	1	-
	Ofloxacin	0.00	0.00–18.43	0	17	3	0
	Norfloxacin	29.70	22.9–37.5	44	148	3	57
	Pefloxacin	**52.38**	0.029–99.76	4	33	2	0
	Nalidixic acid	**80.90**	50.0–98.0	8	9	2	0
Macrolide and related drugs	Clindamycin	27.18	7.9–61.8	44	184	2	97
	Erythromycin	11.90	7.75–17.9	19	159	2	0
Sulfamides	Cotrimoxazole	**73.77**	8.5–98.8	168	310	6	96
Cyclines	Tetracycline	**60.09**	44.75–73.6	177	322	6	26
Phenicols	Chloramphenicol	25.00	47.3–69.0	54	308	6	67
Multidrug resistance		18.00	03.0–63.0	86	237	4	94

CI, confidence interval; *n*, number of resistant strains; *N*, number of strains tested; *I*^2^, heterogeneity.

† , Data in bold indicate significant values (high, exceeding 50%).

### Prevalence of antibiotic-resistant *Escherichia coli* strains

Table 3 shows the overall prevalence of antibiotic resistant *E.coli* strains isolated from dogs in Africa. An analysis of this table shows that the *E. coli* isolates tested were resistant to cefuroxime, cefotaxime, ceftazidime, ceftriaxone and clindamycin, all with 100% of prevalence. These strains were also resistant to kanamycin, ampicillin, amoxicillin, penicillin G, cefalotin, nalidixic acid, gentamicin, cotrimoxazole, tetracycline, doxycycline, chloramphenicol, nitrofurantoin and tylosin with more than 50% of prevalence. In addition, less than 50% of the strains tested were resistant to the other antibiotics evaluated in the selected studies. Beta-lactams, macrolides and related agents, sulfonamides, cyclines, phenicols and nitrofurans are the families in which resistance of *Escherichia coli* strains is most marked. In addition, 98% of isolates are multidrug resistant.

**TABLE 3:** The overall prevalence of antibiotic resistant *Escherichia coli* strains isolated from dogs in Africa.

Families	Antibiotics	Overall prevalence† (%)	95% CI	*n*	*N*	Number of studies	*I* ^2^
Beta-lactams	Ampicillin	**86.49**	71.38–94.26	32	37	3	0
	Amoxicillin	**80.41**	46.21–95.15	279	356	3	94
	Amoxicillin-clavulanic acid	45.11	25.59–66.25	120	224	4	49
	Penicillin G	**99.40**	95.90–99.92	167	168	2	0
	Cefalotin	**83.73**	77.32–88.60	139	166	1	-
	Cefuroxim	**100.00**	97.45–100.00	147	147	1	-
	Ceftriaxone	**100.00**	97.45–100.00	147	147	1	-
	Ceftazidime	**100.00**	74.12–100.00	11	11	1	-
	Cefotaxime	**100.00**	74.12–100.00	11	11	1	-
Aminoglycosides	Streptomycin	40.93	29.04–53.98	88	225	5	52
	Gentamicin	30.54	6.72–72.84	205	391	6	94
	Neomycin	38.46	22.10–57.93	10	26	2	0
	Kanamycin	**57.30**	50.07–64.23	106	185	2	16
	Amikacin	36.75	29.76–44.34	61	166	1	-
Quinolones	Ciprofloxacin	6.05	0.75–35.43	11	225	5	69
	Enrofloxacin	22.46	9.70–43.86	33	182	3	62
	Orbifloxacin	20.99	15.40–27.94	34	162	1	-
	Ofloxacin	8.13	2.23–25.59	13	188	2	90
	Norfloxacin	26.35	9.37–55.31	12	48	5	50
	Pefloxacin	4.79	2.51–8.94	9	188	2	62
	Nalidixique acid	**64.80**	20.95–92.75	14	33	4	37
	Sparfloxacin	14.63	6.73–28.96	6	41	1	-
Macrolide and related drugs	Clindamycin	**100.00**	97.66–100.00	160	160	1	-
	Tylosin	**95.03**	90.38–97.50	153	161	1	-
Sulfamides	Cotrimoxazole	**58.13**	32.74–79.84	88	246	5	86
Cycline	Tetracyclinee	**70.27**	53.87–82.71	26	37	3	0
	Doxycycline	**67.47**	59.98–74.16	112	166	1	-
Phenicols	Chloramphenicol	**71.33**	14.04–97.43	196	332	5	22
Nitrofuranes	Nitrofurantoin	**77.55**	70.10–83.58	114	147	1	-
Multidrug resistance		98.00	81.00–100.00	377	388	5	76

CI, confidence interval; *n*, number of resistant strains; *N*, number of strains tested; *I*^2^, heterogeneity.

† , Data in bold indicate significant values (high, exceeding 50%).

### Prevalence of antibiotic-resistant *Salmonella* strains

The overall prevalence of antibiotic resistant *Salmonella* strains isolated from dogs in Africa is summarised in Table 4. An analysis of this table shows that a significant proportion of the *Salmonella* strains tested were resistant to ampicillin, penicillin G, ceftazidime, cefotaxime, gentamicin, lincomycin, cotrimoxazole, oxytetracycline, streptomycin and vancomycin, with prevalences ranging from 53% to 100%. Regarding sulfamethazine, chloramphenicol, tetracycline, cefalotin, doxycycline and amoxicillin and - clavulanic acid, less than half of the strains tested were resistant. However, none of the *Salmonella* strains tested were resistant to the quinolone antibiotics, particularly norfloxacin, ciprofloxacin and enrofloxacin. The prevalence of multidrug resistant *Salmonella* strains is 53%.

**TABLE 4:** The overall prevalence of antibiotic resistant *Salmonella* strains isolated from dogs in Africa.

Families	Antibiotics	Overall prevalence† (%)	95% CI	Number of strains tested		Number of studies	*I* ^2^
				*n*	*N*		
Beta-lactams	Ampicillin	**52.00**	39.00–64.00	32	62	3	43
	Amoxicillin-clavulanic acid	35.74	19.13–56.66	21	62	3	65
	Penicillin G	93.33	64.80–99.07	14	15	1	-
	Cefalotin	33.33	20.84–48.71	14	42	1	-
	Ceftazidime	100.00	56.55–100.00	5	5	1	-
	Cefotaxime	100.00	56.55–100.00	5	5	1	-
Aminoglycosides	Streptomycin	57.90	32.30–79.86	31	62	3	72
	Gentamicin	80.00	30.90–97.28	4	5	1	-
	Neomycin	50.00	35.32–64.68	21	42	1	-
Quinolones	Ciprofloxacin	0.00	0.00–43.45	0	5	1	-
	Enrofloxacin	0.00	0.00–43.45	0	5	1	-
	Norfloxacin	0.00	0.00–20.39	0	15	1	-
Macrolide and related drugs	Lincomycin	66.67	40.60–85.40	10	15	1	-
Sulfamide	Cotrimoxazole	100.00	56.55–100.00	5	5	1	-
	Sulfamethazine	13.33	3.36–40.54	2	15	1	-
Cyclines	Tetracycline	30.19	19.39–43.74	16	53	2	52
	Doxycycline	33.33	22.38–46.44	19	57	2	0
	Oxytetracycline	59.52	44.26–73.14	25	42	1	-
Phenicols	Chloramphenicol	20.00	6.59–46.98	3	15	1	-
Glycopeptides	Vancomycin	53.33	29.30–75.91	8	15	1	-
Multidrug resistance		31.60	22.40–42.00	30	95	3	81

CI, confidence interval; *n*, number of resistant strains; *N*, number of strains tested; *I*^2^, heterogeneity.

† , Data in bold indicate significant values (high, exceeding 50%).

### Prevalence of antibiotic-resistant *Pseudomonas aeruginosa* strains

Table 5 shows the prevalence of antibiotic-resistant *P. aeruginosa* strains isolated from dogs in Africa. From analysis of this table, all *P. aeruginosa* isolates tested are resistant to ampicillin, amoxicillin, cloxacillin, cephalexin, flucloxacillin, cefuroxime, gentamicin, neomycin, norfloxacin, nalidixic acid, erythromycin, cotrimoxazole and nitrofurantoin. Prevalences of more than 50% in *P. aeruginosa* strains are resistant to amoxicillin and clavulanic acid, penicillin G, carbenicillin, piperacillin, ceftazidime, enrofloxacin, orbifloxacin, lincomycin, tetracycline, doxycycline, chloramphenicol and tylosin. Also, there is a low prevalence of *P. aeruginosa* strains detected to be resistant to imipenem, tobramycin and ciprofloxacin. However, these strains are sensitive to ofloxacin. Resistance of *P. aeruginosa* is marked in all families of antibiotics tested and the prevalence of multidrug resistant strains is 92%.

**TABLE 5:** Prevalence of antibiotic-resistant *Pseudomonas aeruginosa* strains isolated from dogs in Africa.

Families	Antibiotics	Overall prevalence† (%)	95% CI	*n*	*N*	Number of studies	*I* ^2^
Beta-lactams	Ampicillin	**100.00**	72.25–100.00	10	10	2	0
	Amoxicillin	**100.00**	51.01–100.00	4	4	1	-
	Amoxicillin-clavulanic acid	**93.83**	88.91–96.65	152	162	3	0
	Penicillin G	**96.25**	91.91–98.31	154	160	2	0
	Carbenicillin	**92.08**	84.95–95.99	93	101	1	-
	Cloxacillin	**100.00**	51.01–100.00	4	4	1	-
	Cephalexin	**100.00**	51.01–100.00	4	4	1	-
	Flucloxacillin	**100.00**	51.01–100.00	4	4	1	-
	Piperacillin	**86.02**	77.40–91.71	80	93	1	-
	Imipenem	6.00	2.72–12.72	6	100	1	-
	Cefuroxime	**100.00**	51.01–100.00	4	4	1	-
	Ceftazidime	**77.23**	68.05–84.37	78	101	1	-
Aminoglycosides	Streptomycin	**83.33**	36.87–97.72	5	6	1	-
	Gentamicin	**60.00**	0.26–100.00	6	10	2	0
	Neomycin	**100.00**	60.97–100.00	6	6	1	-
	Tobramycin	12.50	7.24–20.73	12	96	1	-
Quinolones	Ciprofloxacin	40.00	0.63–94.10	4	10	2	0
	Enrofloxacin	**74.37**	67.05–80.54	119	160	2	0
	Orbifloxacin	**90.13**	84.28–93.96	137	152	1	-
	Ofloxacin	0.00	0.00–48.99	0	4	1	-
	Norfloxacin	**100.00**	72.25–100.00	10	10	2	0
	Nalidixic acid	**100.00**	60.97–100.00	6	6	1	-
Macrolides and related drugs	Lincomycin	**98.04**	94.10–99.37	150	153	1	-
	Erythromycin	**100.00**	39.58–100.00	4	4	1	-
	Tylosin	**92.86**	87.56–96.00	143	154	1	-
Sulfamides	Cotrimoxazole	**100.00**	72.25–100.00	10	10	2	0
Cyclines	Tetracycline	**90.00**	53.28–98.61	9	10	2	0
	Doxycycline	**87.01**	80.73–91.47	134	154	1	-
Phenicols	Chloramphenicol	**87.34**	1.43–99.82	138	158	3	0
Nitrofuranes	Nitrofurantoine	**100.00**	51.01–100.00	4	4	1	-
Multidrug resistance		**92.25**	87.00–96.00	143	155	1	-

CI, confidence interval; *n*, number of resistant strains; *N*, number of strains tested; *I*^2^, heterogeneity.

† , Data in bold indicate significant values (high, exceeding 50%).

### Prevalence of antibiotic-resistant *Streptococcus pyogenes* strains

Table 6 shows the overall prevalence of antibiotic resistant *S. pyogenes* strains isolated from dogs in Africa. Streptococcus. pyogenes isolates are resistant to ampicillin, amoxicillin, penicillin G, flucloxacillin, cefuroxime, streptomycin, neomycin, nalidixic acid, erythromycin, tetracycline, chloramphenicol and nitrofurantoin with an overall prevalence of 100.00%. A high prevalence of norfloxacin-resistant *S. pyogenes* (87.50%) is also recorded. But these isolates are susceptible to cloxacillin, cephalothin, ciprofloxacin, enrofloxacin and ofloxacin. In addition, resistance in *S. pyogenes* affected all families of antibiotics tested.

**TABLE 6:** The overall prevalence of antibiotic resistant *Streptococcus pyogenes* strains isolated from dogs in Africa.

Families	Antibiotics	Global prevalence (%)	95% CI	*n*	*N*	Number of studies	*I* ^2^
Beta-lactams	Ampicillin	100.00	67.56–100.00	8	8	3	0
	Amoxicillin	100.00	64.57–100.00	7	7	2	0
	Amoxicillin-clavulanic acid	12.50	1.73–53.73	1	8	3	0
	Cloxacillin	0.00	0.00–35.43	0	7	2	0
	Penicillin G	100.00	20.65–100.00	1	1	1	-
	Cefalotin	0.00	0.00–35.43	0	7	2	0
	Flucloxacillin	100.00	64.57–100.00	7	7	2	0
	Cefuroxime	100.00	64.57–100.00	7	7	2	0
Aminoglycoside	Streptomycin	100.00	20.65–100.00	1	1	1	-
	Gentamicin	12.50	1.73–53.73	1	8	3	0
	Neomycin	100.00	20.65–100.00	1	1	1	-
Quinolone	Ciprofloxacin	0.00	0.00–32.44	0	8	3	0
	Enrofloxacin	0.00	0.00–79.35	0	1	1	-
	Ofloxacin	0.00	0.00–35.43	0	7	2	0
	Norfloxacin	87.50	46.27–98.27	7	8	3	0
	Nalidixic acid	100.00	20.65–100.00	1	1	1	-
Macrolides	Erythromycin	100.00	64.57–100.00	7	7	2	0
Sulfamide	Cotrimoxazole	12.50	1.73–53.73	1	8	3	0
Cycline	Tetracycline	100.00	67.56–100.00	8	8	3	0
Phenicols	Chloramphenicol	100.00	67.56–100.00	8	8	3	0
Nitrofuranes	Nitrofurantoine	100.00	64.57–100.00	7	7	2	0

CI, confidence interval; *n*, number of resistant strains; *N*, number of strains tested; *I*^2^, heterogeneity.

### Prevalence of antibiotic resistant coagulase negative *Staphylococcus* strains

The overall prevalence of antibiotic resistant coagulase negative *Staphylococcus* (SCN) strains isolated from dogs in Africa is shown in Table 7. Analysis of this table shows that all isolates tested are resistant to ampicillin, penicillin G, cotrimoxazole and tetracycline. Furthermore, high prevalences of resistant SCN were also recorded for amoxicillin, amoxicillin and clavulanic acid, cefuroxime, nalidixic acid and chloramphenicol. For methicillin, streptomycin, ceftriaxone, gentamicin and enrofloxacin, less than half of the isolates tested were found to be resistant. In addition, isolates were found to be susceptible to ciprofloxacin, norfloxacin and pefloxacin.

**TABLE 7:** The overall prevalence of antibiotic resistant coagulase negative *Staphylococcus* strains isolated from dogs in Africa.

Families	Antibiotics	Overall prevalence† (%)	95% CI	*n*	*N*	Number of studies	*I* ^2^
Beta-lactams	Ampicillin	**100.00**	60.97–100.00	6	6	1	-
	Amoxicillin	**82.35**	57.29–94.20	14	17	1	-
	Amoxicillin-clavulanic acid	**83.33**	36.87–97.72	5	6	1	-
	Penicillin G	**100.00**	60.97–100.00	6	6	1	-
	Methicillin	33.33	8.39–73.19	2	6	1	-
	Cefuroxime	**64.71**	40.41–83.21	11	17	1	-
	Ceftriaxone	35.29	16.79–59.59	6	17	1	-
Aminoglycosides	Streptomycin	26.08	0.00–99.39	6	23	2	0
	Gentamicin	21.74	9.35–42.80	5	23	2	0
	Neomycin	**83.33**	36.87–97.72	5	6	1	-
Quinolones	Ciprofloxacin	0.00	0.00–14.31	0	23	2	0
	Enrofloxacin	16.67	2.28–63.13	1	6	1	-
	Norfloxacin	0.00	0.00–39.03	0	6	1	-
	Pefloxacin	0.00	0.00–18.43	0	17	1	-
	Nalidixic acid	**66.67**	26.81–91.61	4	6	1	-
Sulfamides	Cotrimoxazole	**100.00**	60.97–100.00	6	6	1	-
Cyclines	Tetracycline	**100.00**	60.97–100.00	6	6	1	-
Phenicols	Chloramphenicol	**83.33**	36.87–97.72	5	6	1	-

CI, confidence interval; *n*, number of resistant strains; *N*, number of strains tested; *I*^2^, heterogeneity.

† , Data in bold indicate significant values (high, exceeding 50%).

### Prevalence of antibiotic resistant *Staphylococcus pseudintermedius*

Table 8 shows the overall prevalence of antibiotic-resistant *S. pseudintermedius* strains isolated from dogs in Africa. Analysis of this table shows that high prevalences of antibiotic resistant *S. pseudintermedius* isolates are found only in ampicillin (66.07%), penicillin G (53.19%) and clindamycin (51.79%). On the other hand, these strains are mainly susceptible to tobramycin, teicoplanin, vancomycin and mupirocin. However, a low prevalence of resistant isolates was observed for the other antibiotics tested, with percentages ranging from 1.82% to 29.09%. The prevalence (25.00%) of multidrug resistance was low.

**TABLE 8:** The overall prevalence of antibiotic resistant *Staphylococcus pseudintermedius* strains isolated from dogs in Africa.

Families	Antibiotics	Overall prevalence† (%)	95% CI	*n*	*N*	Number of studies	*I* ^2^
Beta-lactams	Ampicillin	**66.07**	52.83–77.20	37	56	1	-
	Amoxicillin-clavulanic acid	17.86	9.89–30.11	10	56	1	-
	Penicillin G	**53.19**	37.46–68.32	59	111	2	
	Cefalotin	8.93	3.77–19.72	5	56	1	-
Aminoglycoside	Streptomycin	1.82	0.26–11.81	1	55	1	-
	Gentamicin	4.50	0.09–34.83	5	111	2	0
	Kanamycin	5.85	1.49–20.31	8	111	2	72
	Tobramycin	0.00	0.00–6.53	0	55	1	-
	Amikacin	3.60	0.15–25.03	4	111	2	0
Quinolones	Ciprofloxacin	1.82	0.26–11.81	1	55	1	-
	Enrofloxacin	8.93	3.77–19.72	5	56	1	-
	Orbifloxacin	14.29	7.31–26.05	8	56	1	-
	Clindamycin	**51.79**	38.87–64.47	29	56	1	-
Sulfamides	Cotrimoxazole	19.82	13.42–28.27	22	111	2	0
Cyclins	Tetracycline	29.09	18.65–42.33	16	55	1	-
	Doxycyclin	23.21	13.98–35.99	13	56	1	-
Phenicols	Chloramphenicol	7.16	1.15–33.84	11	105	2	83
Macrolides	Tylosin	16.07	8.58–28.09	9	56	1	-
Glycopeptides	Teicoplanin	0.00	0.00–6.53	0	55	1	-
	Vancomycin	0.00	0.00–6.53	0	55	1	-
Fusidic acids	Fusidic acid	7.27	2.76–17.83	4	55	1	-
Mupirocin	Mupirocin	0.00	0.00–6.53	0	55	1	-
Multidrug resistance		24.62	20.00–30.00	82	333	2	31

CI, confidence interval; *n*, number of resistant strains; *N*, number of strains tested; *I*^2^, heterogeneity.

† , Data in bold indicate significant values (high, exceeding 50%).

### Resistance to antibiotics common to the seven strains

**FIGURE 3:**
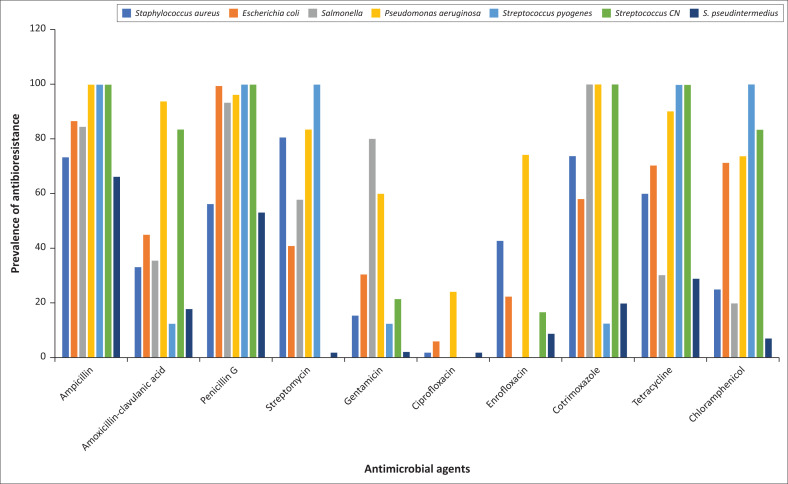
Antibiotic resistance of strains from sampled animals.

Figure 3 summarises the prevalence of the seven bacterial strains according to the antibiotics they have in common. The figure shows that all strains were resistant to ampicillin and penicillin G. With the exception of *P. aeruginosa*, which is resistant to enrofloxacin (74.37%), all strains were sensitive to ciprofloxacin and enrofloxacin. Only *Salmonella* (80.00%) and *P. aeruginosa* (60.00%) were resistant to gentamicin. On the other hand, *P. aeruginosa* (93.83%) and *Staphylococcus* negative coagulase (83.33%) were resistant to amoxicillin and clavulanic acid, whilst for streptomycin and cotrimoxazole, *Staphylococcus* negative coagulase and *S. pseudintermedius* isolates were found to be weakly resistant. *Escherichia coli* isolates were moderately resistant to streptomycin (40.93%) and *S. pyogenes* isolates were weakly resistant to cotrimoxazole (12.5%). All strains were resistant to tetracycline, except *Salmonella* (30.19%) and *S. pseudintermedius* (29.09%). With the exception of *S. aureus* (25.00%), *Salmonella* (20.00%) and *S. pseudintermedius* (7.16%), which were sensitive to chloramphenicol, the other strains were resistant to this antibiotic.

### Prevalence of multidrug resistant strains in dogs

**FIGURE 4:**
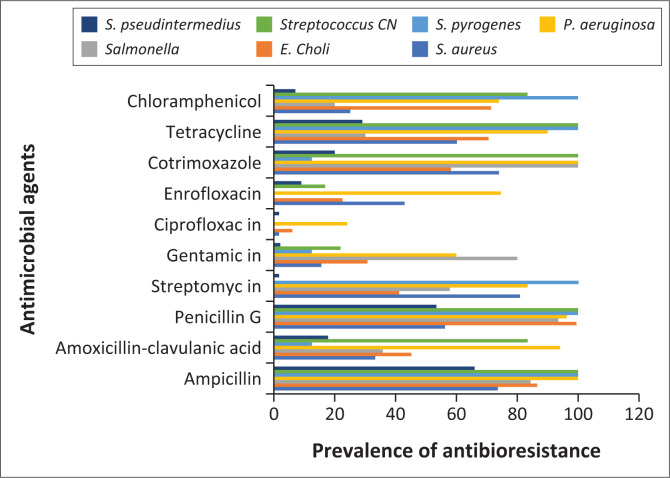
Prevalence of multidrug resistant strains in dogs in Africa.

The prevalences of multidrug resistant strains isolated from dogs in Africa are presented in Figure 4. From the analysis of this table it is obvious that the *E. coli* strain has the highest prevalence of multidrug resistance followed by *Streptococcus CN* first then *P. aeruginosa then S. pyogenes*. In contrast, the prevalence is low for *S. aureus* and *S. pseudintermedius* (Appendix 1).

## Discussion

Antibiotic resistance is a growing phenomenon in African countries and in companion animals, notably dogs. Many antibiotics used in veterinary medicine are similar to those used in human medicine. The present systematic review assessed the overall prevalence of antibiotic-resistant bacterial strains isolated from dogs in Africa and found that many strains were multidrug resistant to most common and current used antibiotics (ampicillin, ampicillin/cloxacillin, tetracycline, penicillin, amoxicillin, cotrimoxazole, chloramphenicol and ciprofloxacin) used in humans (Okpara et al. 2018). The high resistance of *S. aureus* strains to ampicillin found in dogs was also observed in humans (100.0%) and pigs (100.0%) in Tanzania by Katakweba et al. (2016). But for tetracycline, resistance is moderate in humans (45.5%) and pigs (50.0%) in Tanzania, whilst it is high in dogs. In the Democratic Republic of Congo, resistance prevalences of 33.00% – 72.00% against tetracycline, 5.00% – 54.00% against cotrimoxazole, 31.00% against gentamicin, 26.00% – 69.00% against erythromycin and 20.00% – 59.00% against ciprofloxacin have been reported in humans (Lupande-Mwenebitu et al. 2020). Our study showed contrasting findings: whilst the results are similar to those obtained in Europe by Moyaert et al. (2019), the prevalence of penicillin G-resistant isolates in the present analysis (56.23%) is lower than that obtained in Europe (65.20%). Furthermore, strains from the seven studied African countries were resistant to cotrimoxazole (73.77%) and tetracycline (60.09%), whilst those isolated from European countries were found to be sensitive to the same antibiotics, with low resistance prevalences equal to 0.00% and 13.00%, respectively (Moyaert et al. 2019). *Staphylococcus aureus* strains were resistant to nalidixic acid, streptomycin, methicillin, cotrimoxazole, ampicillin, amoxicillin, kanamycin, tetracycline, penicillin G and pefloxacin.

With regard to multidrug resistance, the prevalence in dogs in the present study (18.0%) is lower than that recorded in humans in Nigeria (68.0%) (Ogundipe et al. 2020) and in cats (28.6%) in South Africa (Qekwana et al. 2017).

In this study, *E. coli* strains were resistant to 18 antibiotics. These were cefuroxime, cefotaxime, ceftazidime, ceftriaxone, clindamycin, kanamycin, ampicillin, amoxicillin, penicillin G, cefalotin, nalidixic acid, gentamicin, cotrimoxazole, tetracycline, doxycycline, chloramphenicol, nitrofurantoin and tylosin. These results differ from those obtained in Europe by Moyaert et al. (2019), where *E. coli* strains tested were susceptible to ampicillin, cotrimoxazole and tetracycline, marked by low prevalences of resistance of 36.40%, 21.20% and 18.2%, respectively. However, the sensitivity of *E. coli* strains to orbifloxacin observed in this study is also reported in the study conducted in Europe by Ludwig et al. (2016) where the prevalence of resistant isolates was low, 3.7%. Furthermore, in the meta-analysis study conducted by Emami, Javanmardi and Pirbonyeh (2020), 73.00% of *E. coli* strains isolated from pregnant women in Africa were resistant to ampicillin and 52.00% of *E. coli* strains from Asia. These prevalences are lower than the prevalence in dogs in the present study (86.49%). Compared with gentamicin (30.54%), amoxicillin and clavulanic acid (45.11%) and tetracycline (70.27%), the prevalences of resistant *E. coli* in dogs are higher than those in humans in Africa, which are 23.00%, 44.00% and 60.00%, respectively, but the prevalence of ciprofloxacin resistant *E. coli* in dogs (6.05%) is very low compared with that in humans (26.00%).

Regarding antibiotic resistance of *Salmonella* strains, the results are not in agreement with those obtained in Europe by Bataller et al. (2020) who reported that Salmonella serovars are susceptible to ceftazidime, cefotaxime, gentamicin and cotrimoxazole. Nevertheless, the results obtained for the sensitivity of *Salmonella* strains to ciprofloxacin are similar to those reported by the same authors.

For *P. aeruginosa* strains, the results observed differ from those obtained in the studies conducted in Europe by Ludwig et al. (2016), where the prevalence of *P. aeruginosa* strains resistant to gentamicin and enrofloxacin are low, 18.80% and 18.20%, respectively.

Regarding coagulase negative *Staphylococcus* strains, the results recorded in this study are contrary to those reported by Wedley et al. (2014) who observed the sensitivity of United Kingdom (UK) coagulase negative *Staphylococcus* isolates to cotrimoxazole and tetracycline, with resistance prevalences of 22.50% and 27.50%, respectively. In contrast, methicillin, which is effective on African isolates with a low prevalence of resistance (33.33%), was found to be ineffective on UK strains, with a resistance prevalence equal to 100.00% (Wedley et al. 2014).

As for *S. pseudintermedius*, the result observed for penicillin G activity on isolates in this study is different from that obtained in Europe by Moyaert et al. (2019) where tested *S. pseudintermedius* isolates were susceptible to this antibiotic, with a resistance prevalence equal to 20.0%. However, this result is similar to the one obtained in Japan by Bardiau et al. (2013), but the prevalence recorded in our study for this antibiotic (53.19%) is lower than that observed in Japan (> 95.0%). Compared with tetracycline and enrofloxacin, the susceptibility of *S. pseudintermedius* strains observed in this study is also found in European isolates by Moyaert et al. (2019). For clindamycin, the prevalence obtained (51.79%) is lower than that obtained in work carried out in European and North American regions by Perreten et al. (2010), where the prevalence of resistant strains is equal to 89.3%.

On the other hand, the results for chloramphenicol (7.16%), ciprofloxacin (1.82%), enrofloxacin (8.93%), gentamicin (4.50%), kanamycin (5.85%), streptomycin (1.82%) and tetracycline (29.09%) are contrary to those reported by Perreten et al. (2010) who reported resistance of *S. pseudintermedius* isolates to these antibiotics, with prevalences of 57.30%, 87.40%, 84.50%, 69.90%, 93.20%, 90.30% and 69.90%, respectively. The sensitivity of *S. pseudintermedius* strains from Africa to vancomycin (100%) confirms the results of Perreten et al. In Japan, *S. pseudintermedius* strains were found to be resistant to ciprofloxacin, streptomycin, gentamicin, kanamycin, cotrimoxazole, chloramphenicol and tetracycline, with prevalences ranging from about 70.00% to 100.00% (Bardiau et al. 2013); this differs from the results obtained in the present study.

There are several reasons for the differences in these results: the level of knowledge about antimicrobials and their use, the attitudes of owners towards dog healthcare, the spatial epidemiology of AMR, the level of enforcement of existing legislation on antimicrobials in veterinary medicine, and the conditions of access to veterinary services in given regions. In a study conducted in Nigeria by Okpara et al. (2018), a low level of knowledge on antimicrobials and their use was observed amongst the respondents; 64.4% of the respondents administered antimicrobials to their animals themselves and 60.6% used antimicrobials without veterinary prescriptions. Also, more than half of the respondents (51.5%) had never used veterinary services for their animals whilst 23.1% rarely used them. From the same study, 71.3% of the respondents mentioned that they did not have veterinary practices in their communities.

This can lead to problems such as misdirection of antimicrobials, over- or under-dosing, and poor storage and handling of antimicrobial agents. Regarding the spatial epidemiology of AMR, the study conducted in South Africa by Qekwana et al. (2019) revealed the similarity of prescribing practices amongst veterinarians in the study areas. Given that the realities of African countries differ from those of other continents in several respects, the differences in prevalences may, therefore, be related to differences in prescribing practices. The lack of surveillance programmes on antimicrobial use, as well as the lack of enforcement of existing legislation with appropriate sanctions for violators in African countries may account for the differences in results.

Such high prevalences of multidrug resistant strains amongst the investigated microbial strains constitute a real health problem that could lead to therapeutic failures in these animals and humans, especially their owners. Indeed, the sharing of the resistance genes of these strains between these animals and their owners could be a source of treatment failure of these targets in case of diseases (Feiyang et al. 2021). Educating dog owners about the impact of antibiotic resistance on animals and nearby humans is essential. In addition, appropriate integrated programmes to control AMR in Africa, especially for companion animals, should be implemented.

## Conclusion

Antibiotic resistance is a serious problem worldwide. This phenomenon is increasing in Africa, in pets and particularly in dogs, which are relatively closer companions to humans. All strains investigated are resistant to ampicillin and Penicillin G, but are sensitive to ciprofloxacin. In relation to the other antibiotics, the level of resistance varies according to the strain. Multidrug resistance is much higher in *E. coli, P. aeruginosa* and *Salmonella* strains compared with *S. aureus* and *S. pseudintermedius* strains. The high prevalence of resistance observed for strains of *E. coli, Salmonella, P. aeruginosa, S. pyogenes*, coagulase-negative *Staphylococcus, S. aureus* and *S. pseudintermedius*, which are zoonotic strains, should not be overlooked. As more than one of these bacteria can be found in the same individual and the antibiotics used in animals are practically used in humans for the treatment of infections, it is important to use these antibiotics rationally, so that control of one bacterial strain does not lead to resistance in the other. In view of the multidrug resistance observed in each of these strains, it is important and immediate need to implement measures to prevent the exchange of pathogens between dogs and their owners and to apply good hygienic practices and promote alternatives such as appropriate medicinal plants to combat these different strains in Africa.

## Figures and Tables

**FIGURE 1-A1:**
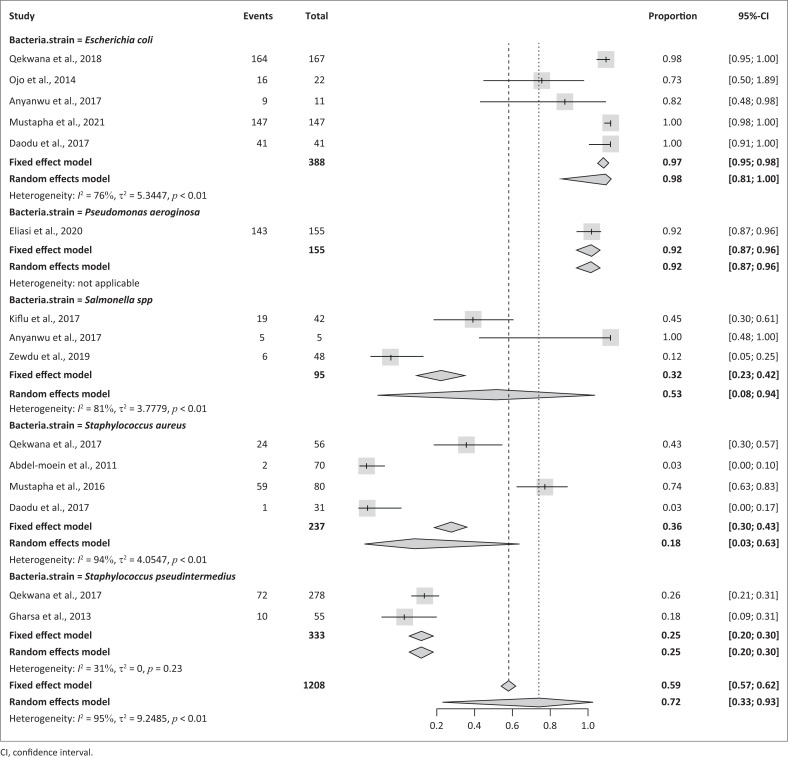
Graph illustrating the multidrug resistance of bacterial strains isolated from dogs in Africa.
